# Copper Mediates Anti-Inflammatory and Antifibrotic Activity of Gleevec in Hepatocellular Carcinoma-Induced Male Rats

**DOI:** 10.1155/2019/9897315

**Published:** 2019-03-03

**Authors:** Iftekhar Hassan, Hossam Ebaid, Ibrahim M. Alhazza, Jameel Al-Tamimi, Shazia Aman, Ahmad M. Abdel-Mageed

**Affiliations:** ^1^Department of Zoology, College of Science, King Saud University, Riyadh 11451, Saudi Arabia; ^2^Department of Biochemistry, J N Medical College and Hospital, Aligarh Muslim University, Aligarh, India; ^3^Department of Zoology, Faculty of Sciences, Minia University, Minia, Egypt

## Abstract

The elevated level of copper is one of the hallmark features of cancer cells in most of the types of cancer. In the present study, this feature has been targeted to investigate if coadministration of exogenous copper (Cu^+^) and its chelating agent like disulfiram (DSF^+^) influence the antineoplastic activity of the anticancer drug, Gleevec (GLV^+^), in hepatocellular carcinoma (HCC)-induced rats via immunomodulation. After the treatment, the level of proinflammatory interleukins (IL-1, 2, 6, and 7), anti-inflammatory interleukin (IL-10) concomitant with transcription factors (NF-kB and TNF-a), and the apoptotic marker (cleaved PARP) was estimated. The cancer-induced group without treatment (CN^+^) demonstrated abnormally elevated level of all proinflammatory cytokines and transcription factors concomitant with a compromised level of cleaved PARP as compared to the control normal (CN^−^). The detailed histological analysis also supported the results exhibiting extensive inflammation and tissue fibrosis confirming the second stage of HCC. Cu+, DSF^+^, and GLV^+^ displayed mild improvement in most of the parameters, but the combination group GLV + Cu^+^ demonstrated remarkable recovery in histology and most of the parameters tended towards the CN^−^ followed by GLV + DSF^+^. Therefore, the management of copper level is critical in realizing the antineoplastic activity of GLV up to its full potential in cancer treatment. These findings will help in improving chemoimmunotherapy and personalized cancer treatment.

## 1. Introduction

Copper (Cu) is one of the essential trace elements for all forms of life. This divalent metal acts as a catalytic cofactor or as an integral component in many vital proteins. Hence, it is an integral structural and functional component in many “cuproproteins” and “cuproenzymes” contributing to diversified orthologs for numerous biological activities including enzymatic catalysis, scavenging of reactive species, erythropoiesis, pigment formation, iron homeostasis, angiogenesis, immunity, cell to cell communication, and even nerve induction [1–3]. Furthermore, Cu plays a very crucial role in COX-mediated ATP generation that vividly illustrates the importance of the metal in the sustenance of life. The metal derives its bioactivity from its excellent redox capability that allows donation and acceptance of the electrons in two valence states as Cu^+^ and Cu^++^ with ease and efficacy in the biological system [4, 5]. This redox property is harnessed in many critical biological functions including enzymatic activity, oxygen transport system, and cell signaling based on oxidation-reduction (redox) reactions in prokaryotes and eukaryotes alike.

However, the redox activity of this metal can be potentially toxic if either its activity is too aggressive or the related biological system is compromised during any disease, metabolic disorder, or infection. In either condition, Cu can catalyze the generation of reactive species/radicals potentially damaging the macromolecules, namely, proteins, lipids, and nucleic acids [6]. Besides, Cu in excess can replace many of the divalent elements like zinc, iron, magnesium, and cobalt that are present in various metalloproteins in the living organisms [7]. Also, a great deal of literature entails the dubious role of Cu in the etiology and proliferation of cancerous cells [8, 9]. Research data from many studies conducted on cancer-induced rodents and cancer patients also shows that copper homeostasis is significantly aberrant and Cu level in serum samples is generally elevated 2-3-fold as compared to their healthy counterparts [10–12]. Intriguingly, this enhanced serum level of Cu has been found to be correlated with the stage of the disease, and it also rebounds to the pretreatment levels during relapse of the disease in the patients on chemotherapy [13, 14]. Despite extensive studies on cancer and malignant cells harboring elevated Cu- level, the exact reason or mechanism has not been elucidated. Therefore, it is still not sure whether cellular transformation to malignancy can lead to accumulation of Cu by 2-4-fold, or the cells adopt such mechanisms to tolerate the burden of tumorigenesis and related oxidative pressure [9, 12, 15, 16]. Also, a study on a mouse model of carcinoma reveals that there was an elevation in the level of copper in serum while its level was decreased in the liver. It entails that the liver plays a central role in mediating the dysregulated copper distribution around the body [17]. Hence, elevated copper has been an attractive drug- target for oncologists and research scientists for over four decades [12, 18, 19].

Gleevec (GLV), also called imatinib mesylate or STI571, is one of the widely administered anticancer drugs against various forms of cancer (chronic myeloid leukemia, gastrointestinal tumors, and systematic mastocytosis) since its discovery in the late 1990s. It has the ability to halt several target kinases (c-Kit, c-Abl, PDGFR-*α*, and EGFR) involved in carcinogenesis and ATP generation as well as regulation of immune cells (macrophages, T cells, NK, and dendritic cells) and inflammatory transcription factors (TNF-*α* and NF-*κ*B) in reversible fashion [20]. The drug enjoys favoritism among the oncologists [20] because it has multiple targeting capabilities with antifibrotic and immunomodulatory properties [21] concomitant with relatively safe adverse effect profile.

**Scheme 1:**
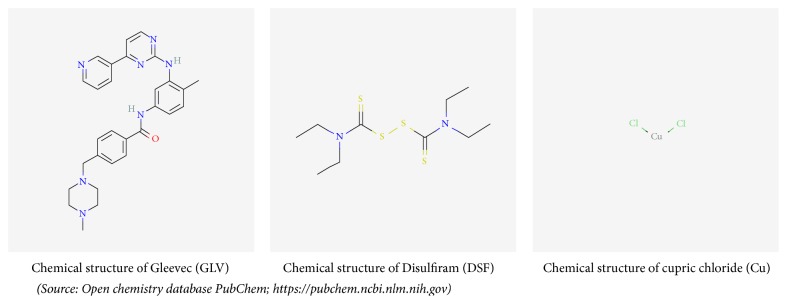


Earlier, we have observed that management of copper level in the cancer cells can enhance the anticancer activity of GLV* in vivo* and* in vitro *[19]. The present study aims to elucidate the possible mechanism involved in the enhanced antineoplastic activity of GLV in hepatocellular carcinoma (HCC) model in Wistar rats coadministered with copper (Cu) and its chelating agent, disulfiram (DSF) (Scheme 1).

## 2. Materials and Methods

### 2.1. Chemicals and Reagents

All the chemicals including copper chloride (CuCl_2_), Gleevec (GLV), and disulfiram (DSF) were purchased from Sigma-Aldrich, St. Louis, MO, USA. All the kits used in the present work were availed from Thermo Fisher Scientific (USA), Abcam (UK), and MyBioSource (Canada). All the other reagents used were bought from different brands with international repute including Merck (Germany), BDH (England), or Sigma-Aldrich (St. Louis, MO, USA).

### 2.2. Methods

#### 2.2.1. Animal Husbandry

Seventy male and adult Wistar rats weighing 110 ± 20 g were purchased from Central Animal House (Department of Pharmacy, King Saud University, Riyadh). All the rats were acclimatized for a week in standard rearing conditions in the Departmental Animal House (Department of Zoology, King Saud University, Riyadh). They were carefully kept under the ethically approved conditions of suitable temperature and humidity with 12 h day: night cycle maintained on a standard rat diet and fresh water* ad labium* in the sufficiently big cages. All the treatment procedures conducted on the animals were performed as per rules of Institutional Ethical Committee of King Saud University, Riyadh.

#### 2.2.2. Treatment with the Test Chemicals

The carefully chosen healthy animals were distributed into seven treatment groups (n = 10). The first group (CN^−^) was taken as a control negative without any treatment. The remaining groups (second to seventh) were administrated with diethylnitrosamine and phenobarbital for two months for the induction of hepatocellular carcinoma in the rats [19]. After two months of cancer induction, blood withdrawn from a retroorbital region of two members of each group was subjected to liver function tests and histopathology to confirm the cancer induction [19, 23]. After confirmation of cancer induction, third, fourth, and fifth group were treated with copper chloride (Cu), Gleevec (GLV), and disulfiram (DSF) at the dose of 25, 35, and 50 mg/kg, respectively, as per our lab standardized treatment strategy [19]. The sixth group was administered with GLV + Cu^+^ while the seventh group was injected with GLV + DSF^+^ at their respective doses. All the groups (third to seventh) were given fresh water* ad labium* during the remaining period of treatment.

#### 2.2.3. Sample Collection

We report that 6 rats died during cancer induction as they might not be able to withstand the treatment. All the remaining rats were sacrificed on a single day after completion of the treatment. Their liver samples were washed in phosphate buffered saline (pH 7.36) and were stored in a deep freezer at -80°C (Eppendorf, UK). Moreover, blood samples were also collected in vacuum tubes (with anticoagulant), and finally, their serum was stored at - 80°C (Eppendorf, UK) after centrifugation at 1200 x g (Eppendorf, UK).

The liver samples from the treated groups were homogenized (Ika, USA) in chilled sodium phosphate buffer (0.1M, pH 7.36) and then centrifuged at 3000 X* g* for 10 min (Eppendorf, Germany). Thus the samples with proper labeling were stored at -80°C until their biochemical analysis.

#### 2.2.4. Measurement of Interleukins (ILs) in Serum Samples

The level of cytokines including IL-1*β* (Catalog number BMS630, Thermo Fisher Scientific Company, USA), IL-2 (Catalog number BMS634, Thermo Fisher Scientific Company, USA), IL-6 (Catalog number BMS625, Thermo Fisher Scientific Company, USA), IL-7 (Catalog number ab100714, Abcam, UK), and IL-10 (Catalog number ab100765, Abcam, UK) was estimated by commercial Elisa kits following the respective manufacturer's instructions.

#### 2.2.5. Assessment of Transcription Factors in Liver Samples

The level of transcription factors- NF*κ*B (Catalog number ab133112, Abcam, UK) and TNF-*α* (Catalog number BMS607-3; Thermo Fisher Scientific Company, USA) was assessed by commercial Elisa kits following the manufacturer's instructions.

#### 2.2.6. Assessment of Apoptotic Marker in Liver Samples

The level of cleaved PARP was assessed by commercial Elisa kits (Thermo Fisher Scientific Company, USA; Catalog number 62219) as per the manufacturer's instructions.

The preparation of the samples from incubation with the primary and secondary antibodies followed by color development under the Elisa based experiments was conducted as per the respective booklet provided with the kits. The optical densities (OD) were measured at 405 nm by Elisa plate reader (Biochrom, UK).

#### 2.2.7. Histopathology of the Liver Samples

The liver samples from all the treatment groups were fixed in 8% formalin for their histopathological analysis. Their paraffin embedding was performed in the tissue blocks (10 x 5 x 3 mm) followed by section cutting (5-7 *µ*m thickness) by a rotary microtome. These prepared sections were stained with Hematoxylin and Eosin dye. Moreover, separate slides were also stained with Mallory Trichrome, Reticulin, and PAS for detection of the collagen deposition in the hepatic tissue sections. Scoring of the fibrosis stage was performed by the Ishak system [22] using the same principles to assess the stage of inflammation and fibrosis of the samples. The stage depends on the amount of fibrous tissue in a liver sample. The higher Ishak scores depend on architectural changes and degree of nodularity rather than the amount of fibrous tissue. The lower scores are dependent on the amount of portal tract collagen because portal tracts can be considerably expanded by inflammatory infiltrates as well. The sections were observed under a light microscope (Leica, Germany) and their photomicrographs were captured at the magnification of 400 X by the camera (Leica, Germany) attached to it.

### 2.3. Statistical Analysis

All the data has been expressed as mean ± SEM analyzed by GraphPad Prism 5 software. The data were analyzed by one-way ANOVA with Tukey's post hoc selecting p-value < 0.05 as statistically significant with the help of the software. The number of asterisk marks *∗* and # indicates significance difference from negative control (group CN^−^) and positive control (group CN^+^) in* in vivo* studies as calculated by Student's t-test. The asterisk marks *∗∗* and ## mean p< 0.01, while *∗∗∗* and ### mean p< 0.001. Minor fluctuations were observed upon repetition of the experiments as indicated in the statistical analysis.

## 3. Results

We conducted our experiments to estimate if the Cu-mediated immunomodulation can enhance the antineoplastic activity of the GLV drug in liver cancer murine models. All the values have been compared to CN^−^ in the result section.

### 3.1. Effect on Cytokines

#### 3.1.1. Effect on IL-1*β*

**Figure 1:**
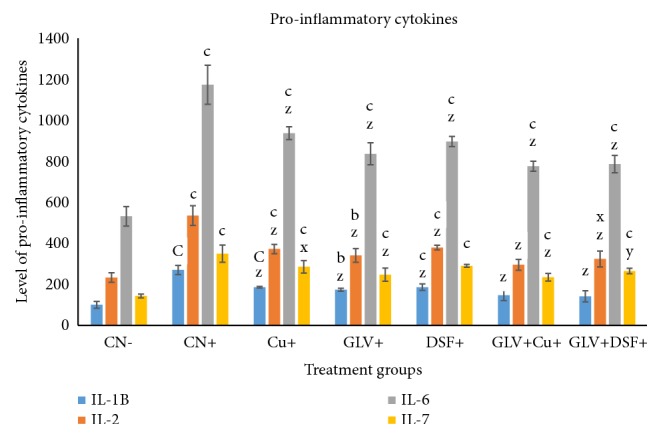
Level (in pg/ml) of proinflammatory cytokines, IL-1, 2, 6, and 7, in serum samples of the indicated groups. The values are mean ± SEM of three independent experiments. a, b, and c mean statistically significant from the control, CN, at p≤ 0.5, 0.05, and 0.005, while x, y, and z mean statistically significant from control positive at p≤ 0.5, 0.05, and 0.005.

This is an important proinflammatory cytokine that increases during inflammation and various diseases and carcinogenesis. Cancer-induced rats without treatment, CN^+^, showed 2.7-fold increase in its level as compared to the control, CN^−^. However, Cu+, GLV^+^, and DSF^+^ displayed elevation in its level by 1.86-, 1.74-, and 1.86-fold concerning CN^−^. Among the combination groups, GLV+CU^+^ and GLV+DSF^+^ demonstrated an increase in its level by 1.46- and 1.41-fold compared to CN^−^ (Figure 1).

#### 3.1.2. Effect on IL-2

This proinflammatory cytokine was increased in CN^+^ by 2.29-fold while Cu^+^, GLV^+^, and DSF^+^ showed its increase by 1.60-, 1.46-, and 1.63-fold as compared to CN^−^. GLV + Cu^+^ and GLV + DSF^+^ exhibited its enhanced level by 1.26- and 1.39-fold with respect to CN^−^ (Figure 1).

#### 3.1.3. Effect on IL-6

CN^+^ showed 2.2-fold increase in its level as compared to the control, CN^−^. Cu^+^, GLV^+^, and DSF^+^ displayed elevation in its level by 1.76-, 1.57-, and 1.68-fold concerning CN^−^. Among the combination groups, GLV + CU^+^ and GLV + DSF^+^ demonstrated 1.46- and 1.47-fold increase in its level in comparison to CN^−^ (Figure 1).

#### 3.1.4. Effect on IL-7

CN^+^ demonstrated an increase in its level by 2.44-fold while Cu^+^, GLV^+^, and DSF^+^ exhibited an elevation in its level by 2-, 1.72-, and 2.02-fold as compared to CN^−^. The combination groups GLV + Cu^+^ and GLV + DSF^+^ showed its increase by 1.69- and 1.84-fold by CN^−^ (Figure 1).

#### 3.1.5. Effect on IL-10

**Figure 2:**
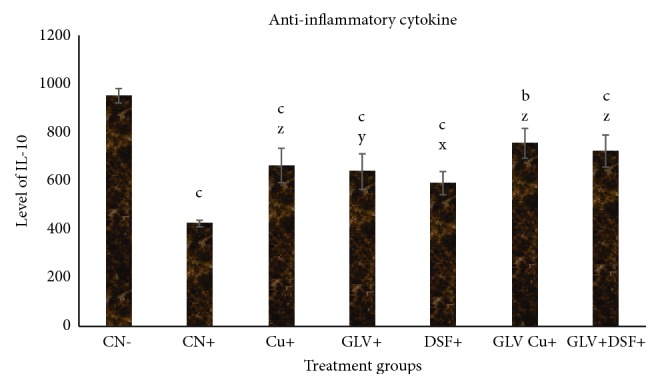
Level (in pg/ml) of anti-inflammatory cytokines, IL-10, in serum samples of the indicated groups. The values are mean ± SEM of three independent experiments. a, b, and c mean statistically significant from the control, CN, at p≤ 0.5, 0.05, and 0.005, while x, y, and z mean statistically significant from control positive at p≤ 0.5, 0.05, and 0.005.

The principal key role of IL-10 is to limit and terminate inflammatory responses. Hence, its level decreases in the case of extensive inflammation and injury while it increases during ceasing of inflammation and tissue repair. Its level was compromised by 55.24% in CN^+^ while it was decreased by 30.05%, 32.69%, and 37.66% in Cu^+^, GLV^+^, and DSF^+^ as compared to CN^−^. GLV + Cu^+^ and GLV + DSF^+^ exhibited decrease in its level by 20.65% and 23.83% with respect to CN^−^ (Figure 2).

#### 3.1.6. Effect on Transcription Factors

In the present study, NF-*κ*B and TNF-*α* were chosen as transcription factors to assess the extent of inflammation, fibrosis, and carcinogenesis.

#### 3.1.7. Effect of NF- *κ*B

**Figure 3:**
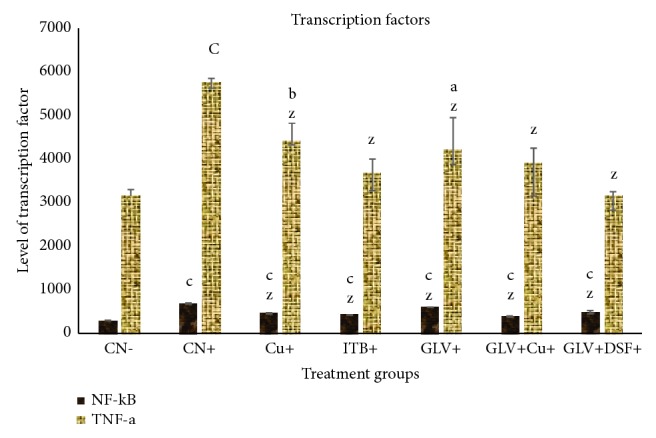
Level (in ng/ml) of transcription factors, NF-*κ*B and TNF-*α*, in liver samples of the indicated groups. The values are mean ± SEM of three independent experiments. a, b, and c mean statistically significant from the control, CN, at p≤ 0.5, 0.05, and 0.005, while x, y, and z mean statistically significant from control positive at p≤ 0.5, 0.05, and 0.005.

CN^+^ exhibited an increase in its level by 2.23-fold while Cu^+^, GLV^+^, and DSF^+^ showed an increase in its level by 1.52-, 1.41-, and 1.97-fold as compared to CN^−^. GLV + Cu^+^ and GLV + DSF^+^ demonstrated an increase in its level by 1.26- and 1.59-fold with respect to CN^−^ (Figure 3).

#### 3.1.8. Effect of TNF-*α*

CN^+^ showed an increase in its level by 1.82-fold while Cu^+^, GLV^+^, and DSF^+^ showed an increase in its level by 1.39-, 1.16-, and 1.33-fold as compared to CN^−^. GLV + Cu^+^ and GLV + DSF^+^ demonstrated an increase in its level by 1.24- and 1.003-fold concerning CN^−^ (Figure 3).

#### 3.1.9. Effect on Cleaved PARP

**Figure 4:**
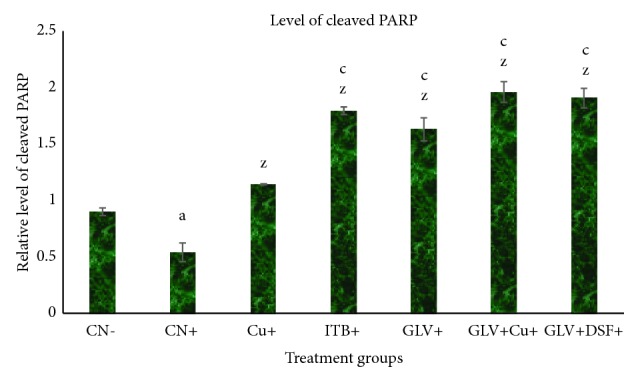
Relative level of the apoptotic marker, cleaved PARP, in liver samples of the indicated groups. The values are mean ± SEM of three independent experiments. a, b, and c mean statistically significant from the control, CN, at p≤ 0.5, 0.05, and 0.005, while x, y, and z mean statistically significant from control positive at p≤ 0.5, 0.05, and 0.005.

The cleaved PARP is an important marker to assess and confirm the progression of apoptosis. PARP14 is involved in normal immune function through the IL-4 signaling pathway and is a prosurvival factor in multiple myeloma and hepatocellular carcinoma. Its level was decreased in CN^+^ by 0.6-fold while it was increased by 1.26-, 1.98-, and 1.81-fold in Cu^+^, GLV^+^, and DSF^+^, respectively as compared to CN^−^. GLV + Cu^+^ and GLV + DSF^+^ demonstrated an increase in its level by 2.17- and 2.12-fold in comparison to CN^−^ (Figure 4).

#### 3.1.10. Effect on Histology of the Liver Samples

**Figure 5:**
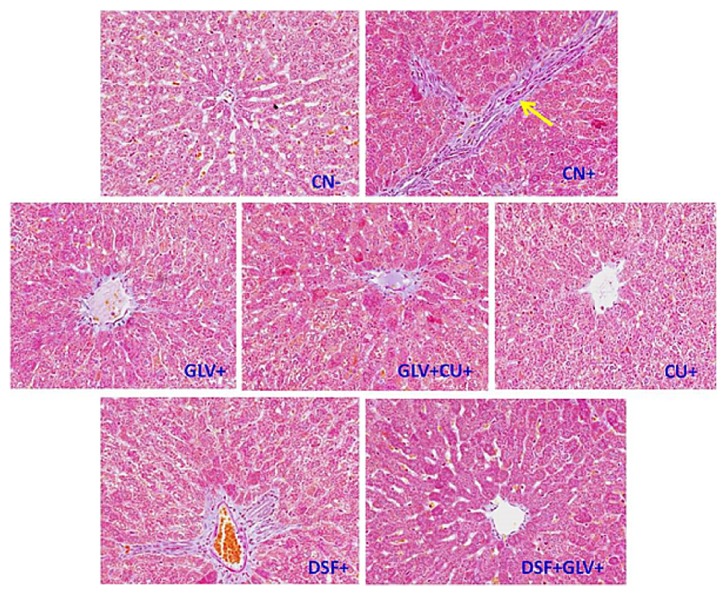
Histological assessment of liver fibrosis in control (CN-) and experimentally induced liver cancer (CN+) rats and the effects of the CU+, ITB, and DSF and the combination of ITB+CU+ and ITB+DSF+ on collagen deposition. The extent of matrix deposition (yellow arrow) was measured by Masson's trichrome staining of liver tissue sections from different rat groups.

Histopathological changes of collagen deposition were quite obvious in the experimentally induced liver cancer (CN^+^) rats without any treatment. Their section revealed the extensive accumulation of connective tissue resulting in the formation of continuous interlobular septa besides noticeable alterations and dilations in the central vein with pronounced signs of inflammation as compared to the normal control (CN^−^). Also, the HCC rats treated with Cu and GLV demonstrated the less histological destruction of the liver architecture with moderate fibrosis and inflammation except for the DSF^+^ group which exhibited additional collagen deposition to a moderate level, particularly around the central veins. However, the combination groups- GLV + Cu^+^ and GLV + DSF^+^ displayed many normally recovering histological features that were quite comparable to the CN^−^ (Figure 5; Table 1).

**Figure 6:**
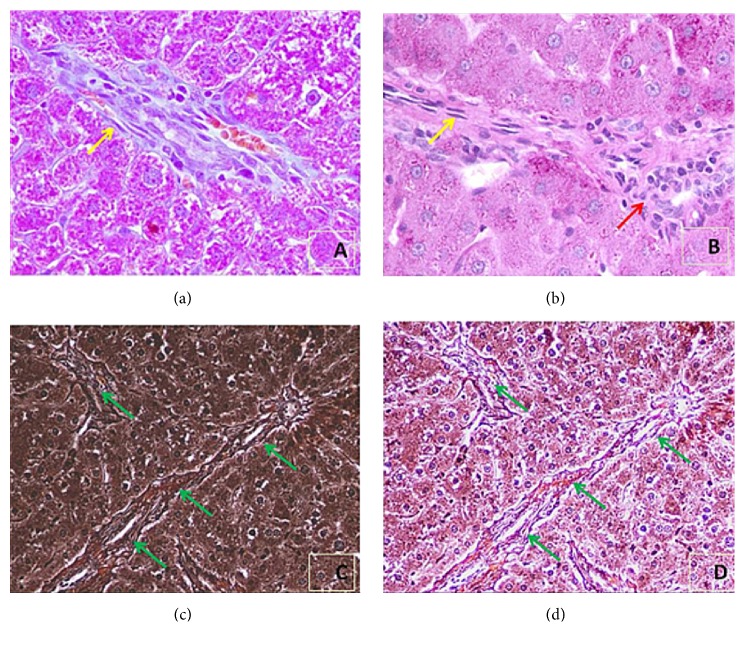
Histological assessment of liver fibrosis in the experimentally induced liver cancer (CN+) rats. The extent of matrix deposition (yellow arrow) was measured by Masson's trichrome ((a); X400) to show fibroblasts (yellow arrow), the periodic acid Schiff (PAS) stain ((b) X400) to show fibroblast (yellow arrow) and infiltrated inflammatory cells (red arrow), and reticulin ((c), (d) different microscopic filters; X200) to show reticular fibers deposition (green arrows) in liver tissue sections from only the second group (CN+).

The degree of liver fibrosis is one of the most important diagnostic and prognostic assessments in chronic liver disease. Clinical manifestations of liver disease and liver dysfunction accompany architectural changes of the liver parenchyma that are considered as a result of advanced stages of liver fibrosis. Thus, we next assessed the stage of fibrosis in the nontreated HCC-induced rats (CN^+^) experimentally induced with liver cancer relative to the treated HCC rats with three histochemical stains. Masson trichome stain showed the collagen deposit into the hepatic tissues of the cancerous rats (CN^+^) in comparison to the control rats (Figure 5).  Figure 6(a) showed a group of proliferated fibroblasts surrounded with a distorted hepatic tissue with narrow sinusoids and altered hepatic chords. Besides, severe damage due to excessive infiltration of inflammatory cells was also a remarked feature in the group (Figure 6(b)). Reticular fiber is comprised of one or more types of thin and delicate strands of collagen III. These strands build a cellular network providing additional support to the network. In the liver, reticulin fibers, however, are present as part of its extracellular matrix. The stain helps in the assessment of the architecture of the hepatic plates, such as expansion in regeneration in neoplastic conditions but collapse of the reticulin framework in the necrotic state. Thus, Figures 6(c) and 6(d) revealed that reticulin fibers are vividly histologically altered through the whole liver section of CN^+^. These features confirm that liver damage is extensive with severe histological alteration as well as related inflammation. Ishak histology rating system was applied in the histological analysis of the present study. According to stage components of this system, hepatic tissues of the CN+ revealed severe damage up to grade 4 (Table 1). However, rats from combination groups GLV + DSF^+^ and GLV + Cu^+^ showed prominently enhanced antifibrotic activity as compared to GLV^+^ rats.

In nut-shell, the present study demonstrates that Cu and DSF decrease the level of proinflammatory cytokines (IL-1*β*, 2, 6, and 7) and transcription factors (NF-*κ*B and TNF-*α*) concomitant with an increase in the level of an anti-inflammatory cytokine (IL-10) and apoptotic marker (cleaved PARP) in HCC rats (Figures 1–4). These factors genuinely contribute to the declination of the extent of inflammation and tissue fibrosis in the combination treated groups (GLV+ Cu and GLV+ DSF) as confirmed by the histopathological analysis. Both the adjuvants (Cu and DSF) enhance the antineoplastic activity of GLV significantly by immunomodulation and apoptosis induction selectively.

## 4. Discussion

Hepatocellular carcinoma or liver cancer is one of the most common forms of cancer. It claims the life of around 600,000 people around the world each year [24]. The disease is slowly progressing with unclear etiology and causative agents/factors. The exact mechanism is not elucidated yet despite extensive research and advancement in the field. Because of the lack of an exact mechanism of pathogenesis of this disease, the effective therapy is elusive. Earlier, we have shown that the restrained manipulation of endogenous copper level in tumor cells orchestrates the redox and molecular status inside the cells that favor the induction of apoptosis [19]. The present study aims to investigate if management of endogenous copper can assist GLV in ceasing carcinogenesis by amelioration of inflammation and fibrosis in HCC rats.

The histological evaluation with the employed treatment strategy in the present study indicates that the cancer induction could be achieved until the second stage of tissue fibrosis. All the findings from current work show that the free radicals (ROS and NOS) and associated inflammation, as well as fibrosis, are the key events during the development of the moderate degree of hepatocellular carcinoma in the treated rats [25]. With administration of the carcinogen (DEN) and promoter (PB), both the agents accumulate in the target organ and elicit the free radicals in its vicinity. These radicals attack the cellular macromolecules and disrupt structural integrity resulting in stage-wise hepatic necroinflammation and liver fibrogenesis followed by low- to high-grade dysplastic nodules [26]. The activation of inflammatory response triggers the release of soluble immune molecules including cytokines, chemokines, ROS, matrix proteinases, or vascular epithelial growth factor (VEGF) by the macrophages and mast cells. They further activate the recruitment as well as infiltration of leukocytes at the site of tissue injury in the target organ. Besides, these events upregulate NF-*κ*B by ROS mediation which in turn induces various proinflammatory cytokines, such as IL-1*β*, TNF-*α*, and IL-6 [27–29]. Overproduction of proinflammatory factors is likely to contribute to the elevation of the inflammatory response leading to partial organ malfunction or complete organ failure and other pathological complications observed in many other inflammatory diseases including cancer [30]. The present work depicts all the events by the persistence and continuation of inflammation and hepatic fibrosis in the target tissue. The progressive hepatic fibrosis later develops as cirrhosis and liver cancer in extreme abrogated form [31]. The cancer induction strategy with DEN-PB occurs in three stages: inflammation (2-6 weeks), hepatic fibrosis (6-12 weeks), and finally full-blown stage of the hepatocellular carcinoma (14-20 weeks) [19]. The current results on cytokines and transcription factors as well as the detailed histological analysis in the present work imply that the HCC could reach up to the fibrosis stage with implemented cancer induction method.

In the inflammatory stage of HCC, antibodies, proinflammatory cytokines, macrophages, and dendritic cells play a vital role in the process of pathogenesis leading to tissue injury [32]. It is a physiological defense mechanism of the body against the injuries and toxic abuses that abrogate into tissue damage and infection if not adequately addressed by the immune system [33]. The infiltration of inflammatory cells, if prolonged, can exasperate into fibrosis through increased generation of ROS via production of profibrotic cytokines and growth factors [21]. Herein, the present research demonstrates that the carcinogen triggered all the critical proinflammatory cytokines (IL-1, 2, 6, and 7) as well as transcription factors (NF-kB and TNF-a) in the target organ. This heightened aggression of the immune system causes extensive inflammation and fibrosis in the liver as evidenced in the histopathological examination. Hence, inflammation and fibrosis can be viewed as a continuum of events within the framework of tissue defense, repair, and regeneration in the present work [34]. Once the tissue damage occurs, it recruits and activates a variety of different cell types of the innate and adaptive immune system that consequently turn into inflammation. The immunological mediators released from T cells, monocytes/macrophages, innate lymphoid cells, basophils, and eosinophils also have both pro- and antifibrotic properties. Also, mesenchymal fibroblasts and other cell types especially infiltrating hematopoietic cells also produce extracellular matrix proteins [35]. Besides the Kupffer cells as resident macrophages in the liver, the combined activity of inflammatory cells including infiltrating macrophages, T lymphocytes, neutrophils, and DCs contributes to liver inflammation. Furthermore, these inflammatory cells activate hepatic stellate cells, which are the major source of myofibroblasts in the liver [36]. Herein, the histological sections show narrowing of the sinusoids as well as the appearance of many large sized Kupffer cells that proves the fibrosis stage of the disease.

**Figure 7:**
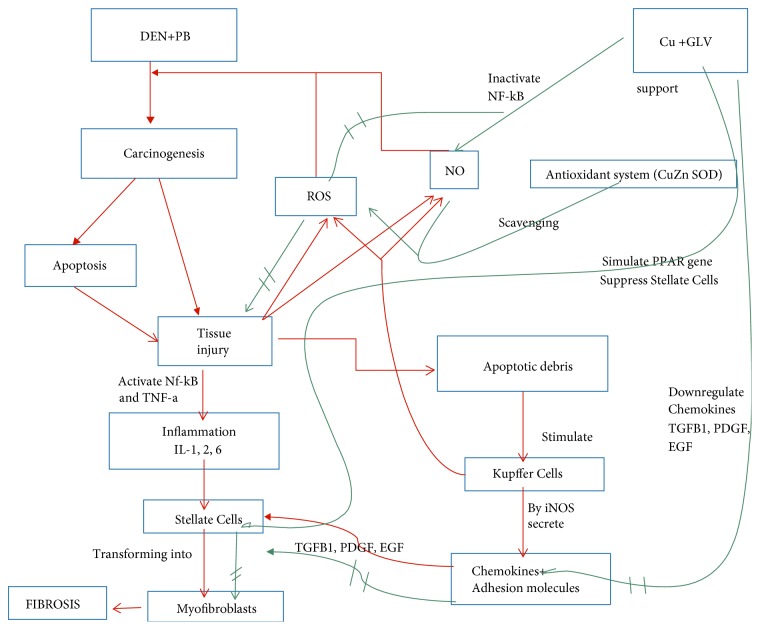
A suggested diagram showing the possible mechanism of the fibrosis stage observed in the liver tissue. The carcinogen-induced hepatic inflammation post-secretion of immunomediators can trigger fibrogenesis by inducing programmed cell death in the hepatocytes, while apoptotic bodies TGF*β*1, PDGF, EGF, etc. can activate nascent hepatic stellate cells transforming them into myofibroblasts. On the other hand, Cu mediates the downregulation of NF-*κ*B and TNF-*α* and growth factors leading to the suppression of the inflammatory cascade and suppression of stellate cells in the treated rats.

Moreover, the carcinogen or virus-induced hepatic inflammation mechanisms can trigger fibrogenesis by inducing programmed cell death in the hepatocytes [37]. The apoptotic bodies can activate nascent hepatic stellate cells transforming them into myofibroblasts under the influence of transforming growth factor beta 1 (TGF*β*1), platelet-derived growth factor (PDGF), and endothelial growth factor (EGF). The apoptotic debris can further incite the Kupffer cells to generate reactive oxygen species (ROS) as well as nitric oxide (NO) by inducible nitric oxide synthase (iNOS). These reactive species, in turn, can increase hepatocyte apoptosis concomitant with activation of hepatic stellate cells. Then, the Kupffer cells can also stimulate the cellular machinery for further ROS generation besides cytokines, and chemokines that can attribute to the transformation of quiescent hepatic stellate cells to myofibroblasts. They can further promote inflammation in hepatic tissues by enhancing the level of proinflammatory cytokines along with adhesion molecules, activated T lymphocytes, and natural killer T (NKT) cells. Also, the myofibroblast activity causes downregulation of antiapoptotic genes that can trigger apoptosis induction (Figure 7). On the contrary, the concurrent expression of the peroxisome proliferator-activated receptor gamma (PPAR*γ*) gene in the same environment can inactivate the hepatic stellate cells that can desensitize them for any growth factors (Czaja et al., 2014). This is one of the most valid explanations of halting the liver carcinogenesis via ceasing the inflammation and progressive tissue fibrosis by GLV with Cu and DSF in the present study.

The present study shows that Cu mediates the downregulation of NF-*κ*B and TNF-*α* leading to the suppression of the inflammatory cascade in the treated rats. TNF-*α* is a key mediator of immune and inflammatory responses that control the expression of the inflammatory gene network. Hence, supplementation of Cu seems to exert broad anti-inflammatory effects in cancer rats leading to hemodynamic performance and the following organ functions as evidenced in the current study. Thus, the amelioration effect of the hepatic tissues by Cu seemed to be mediated by the blocking of the proinflammatory cytokines through inhibition of NF-*κ*B. On the contrary, the anti-inflammatory effect of DSF was compromised by halting the transport of NF-*κ*B from cytosol to the nucleus in the target cells [38]. This might be the reason for the pronounced amelioration of hepatic tissue damage and dysfunction by Cu as compared to DSF in this study.

Intriguingly, the present study shows that Cu seems to have stronger anti-inflammatory and antifibrotic properties as compared to DSF when coadministered with the drug. The present finding is contrary to our previous report that DSF was proposed as stronger apoptosis inducer as compared to Cu when administered with GLV [19]. A great amount of literature shows that the complex of Cu with DSF or another chelating agent like tetrathiomolybdate (TTM) is stronger proteasome inhibitor and apoptosis inducer as compared to DSF alone [18, 39]. Observing the differential antineoplastic efficacy with GLV somehow indicates that both GLV-Cu and GLV-DSF follow the different mode of action in the cancer cells in comparison to the normal ones. One of the key differences is that Cu might cease inflammation by elevating the antioxidant enzyme series led by Cu-Zn-SOD while DSF induces apoptosis by decreasing the GSH:GSSG ratio and mitochondrial membrane potential [19, 40]. It is speculative that Cu might cease HCC-induced inflammation and fibrosis by nullifying the intracellular ROS level while DSF might do the same effect by elevating oxidative stress or by inverting the action of concurrent endogenous Cu level. It seems the net effect of both the agents is potentiating the antineoplastic activity of GLV via downregulation of proinflammatory cytokines and transcription factors and promoting antifibrotic effects in the present study. Despite all these, the level of the anti-inflammatory cytokine, IL-10, and prominent apoptosis marker, cleaved PARP, in GLV+ Cu^+^ was found higher than GLV+ DSF^+^. Both these factors favor attributing to better efficacy in ceasing the inflammation and related fibrosis by the combination GLV+ Cu^+^ as compared to GLV+ DSF^+^.

It is also possible that Cu might enhance either expression or efficacy of the organic cationic transporters (ATP7A and ATP7B) in some unknown way that might facilitate the drug influx in the cancer cells [41–43]. On the other hand, DSF decreases the intracellular Cu in such cells that might hinder a higher influx of the drug. Nevertheless, the cancer cells have an abundance of Cu, so DSF alone cannot inhibit the drug uptake completely. Hence, most of the previous studies show that complex of DSF-Cu is more effective antineoplastic adjuvant with established drugs like GLV and cisplatin as compared to DSF alone [18, 39].

It is also speculative that the elevation of endogenous Cu in cancer cells might be the defense strategy by triggering the immune system via angiogenesis in the transformed tissues [19]. However, somewhere in between angiogenesis and mounting of immune response, a condition arises that facilitates the cancer cells to evade from natural cell death by apoptosis; instead they face aggression of immune system consequently leading to systemic inflammation, recruitment of immune cells, and finally abrogating into fibrosis. However, the Cu supplementation can cease the assault of the immune system by triggering the innate immunity or by orchestrating the redox status that is more favorable for apoptosis induction. The conventional treatment regimens based on immunosuppressive, anti-inflammatory, and antiviral agents can weaken hepatic fibrosis in the patients as their responses are commonly incomplete and inconsistent in efficacy. However, antifibrotic agents seem to have more efficiency as compared to the top anti-inflammatory agents with improved outcomes as the adjunctive therapies (Czaja 2017). Here, the immune-mediated responses may activate hepatic stellate cells by increasing oxidative stress within hepatocytes in the liver cancer-induced rats. In between, angiotensin can be synthesized by these activated hepatic stellate cells that further promote the production of ROS and associated damage (Czaja 2017).

## 5. Conclusion

Hepatic inflammation and tissue fibrosis during induction and progression of HCC can be stopped or reversed by either nullifying the etiologic agent or halting the triggered pathogenesis mechanism of liver damage and related toxic insults. Both Cu and DSF are excellent adjuvants to GLV in ceasing the carcinogenesis by downregulating the proinflammatory cytokines and transcription factors concomitant with a marked elevation in the proapoptotic factors in HCC model rats. However, Cu is more suitable in facilitating the anti-inflammatory and antifibrotic activity of GLV as compared to DSF in the HCC-induced rats. Hence, the management of Cu level in GLV based chemotherapy is one of the keys for achieving higher efficacy of the drug during cancer treatment.

## Figures and Tables

**Table 1:** Stage component of the Ishak system [22] assesses fibrosis in seven categories, ranging from normal (0) to cirrhosis (7) in control and different treated groups.

Groups	CN-	CN+	ITB	ITB+CU+	CU+	DSF+	DSF+ITB+
Stage of fibrosis	0	4	1	1	1	2	1

## Data Availability

The data used to support the findings of this study are included within the article.
